# Everybody's Page

**Published:** 1891-07-04

**Authors:** 


### EVERYBODY'S PAGE.

THERE is a man in New York who makes a handsome living by regulating self-regulating clocks.

MONS. RAOUL PICTET'S new method of preparing crystallised chloroform for commercial use should prove a highly useful discovery if, as is claimed for it, it cannot in this form undergo any changes or contain any impurities.

ACCORDING to the *National Druggist*, M. Lavrand has found that pills of iodide of iron administered either alone or with phosphide of zinc are an efficient remedy to arrest or prevent lead poisoning in those who work in white lead.

A COMBINATION of soda and naphthol, to which the euphonious name of microcidine has been assigned, is claimed by Berlioz to possess strong antiseptic powers employed in solutions of three or five parts to one thousand. It is freely soluble in water, tasteless, and odourless, and is practically devoid of toxic effects.

WE have been so busy over our efforts to perfect the educational system of the country, that in our hurry we seem to have forgotten altogether the health of our children. The recent disclosures, through the agency of the Education Department, as to the "disgraceful condition" in respect to sanitary matters of some of our Board Schools, will, it is hoped, arouse a little more vigilance in this direction for the future.

IN Russia the schoolmaster appears to be very much abroad and the tavern keeper at home. In the entire Porhhofski district, says M. G. P. Sazonoff, £30 a year are spent in schools, six cantons contributing small sums to this total, and the remaining twenty-three districts nothing at all. In several of the villages there is not a person able to read or write, but there are seven hundred taverns with a yearly turnover of two million roubles. "A ha'penny worth of bread to so much sack."

SHADE of the illustrious Rarey! How our forefathers would shudder in their graves if they knew that we are pressing steam into our service for the purpose of training horses! We shall shortly hear that training lap dogs in their tricks by electricity is a favourite pastime with old maiden ladies.

IN a momentary outburst of inspired and poetic eloquence a short time ago, the Home Secretary compared the policeman of to-day, in his sober blue uniform, fulfilling his duty in an unassumed manner, to the knights errant in olden days. It was tantalising of him, to say the least of it, not to throw a little more light upon the resemblance between them, and not to inform the public, whom he was addressing, if the knights errant of old went about shod with boots as dainty and *noiseless* as those which adorn the feet of the modern guardian of the peace—much to the delight of the *fin de siècle* burglar.

A GENERAL deduction from the statistics of the United States census of 1890 points to the fact that residence in the States is exerting an unfavourable influence on the vitality of the Jews. The death-rate amongst them, though remarkably low, compared with that of the rest of the population, is increasing and the birth-rate is diminishing. The marriage-rate is also very low. The factors that militate against their increase in America do not exercise the same influence apparently in Europe. Some fifteen years ago Dr. B. W. Richardson, in writing on the Jews of Germany, drew attention to the fact that the mortality from cholera amongst them is so slight that the fact of its existence is even disputed. "Facts show," he wrote, "that from some cause or causes the Jewish race presents an endurance against disease that does not belong to other portions of the civilized communities. Among infant mortality 10 per cent. die among the Jews, and among other denominations 14 per cent. That the average duration of life of the Jew is 48; of others, only 36. That a quarter of the Jews live beyond 71 years; and of others, 59 years and 10 months."

